# UTILIZING TECHNOLOGY AND INTEGRATED SERVICES TO SUPPORT FAMILY CAREGIVERS IN RURAL COMMUNITIES

**DOI:** 10.1093/geroni/igad104.2015

**Published:** 2023-12-21

**Authors:** Skye Satz, Quinn White, Laura Gallagher Watkin

**Affiliations:** CRIS Healthy Aging, Champaign, Illinois, United States; CRIS Healthy Aging, Danville, Illinois, United States; CRIS Healthy Aging, Champaign, Illinois, United States

## Abstract

CRIS Healthy Aging is a non-profit organization in East Central Illinois that serves older adults who are often lower-income and/or living in a rural community. CRIS’ mission is to keep older adults safe, healthy, and independent in their own homes for as long as possible. CRIS’ Memory Care Program (MCP) expanded upon the evidence-based STAR-Caregivers (STAR-C) program. In addition to administering traditional STAR-C training, CRIS provides caregiving dyads with more integrated services, including (1) implementation of technology like the Amazon Alexa Echo Show devices and OneClick video conferencing for older adults, (2) peer support and education opportunities including Memory Café, Stress-Busting Program for Family Caregivers, and the NCOA Aging Mastery Program, as well as (3) providing financial assistance, respite, and additional aging services. In the past 4 years, the CRIS MCP, funded by a federal ADPI grant through ACL, has served approximately 200 caregiving dyads, and collected pre- and post-treatment survey data from a cohort of 52. Those served consistently reported that the STAR-C program was extremely helpful for them. Most importantly, caregivers reported significant decreases in behavior frequency (t=14.75, p<0.01) and severity of personal stress response (t-21.51, p<0.01). Compared to baseline, caregivers also reported feeling less resentful (p=0.005), less socially isolated (p>0.05, however qualitative data supports downward trend), and saw improvements to quality of life (t=-1.77, p<0.042). Ongoing barriers to service in these rural and low-income communities include a global pandemic, misinformation, fear of the unknown, family unwillingness to participate, and financial concerns.

